# Serum protein triplet TGF-β1, TIMP-1, and YKL-40 serve as diagnostic and prognostic profile for astrocytoma

**DOI:** 10.1038/s41598-021-92328-3

**Published:** 2021-06-23

**Authors:** Rūta Urbanavičiūtė, Rūta Zabitaitė, Algimantas Kriščiukaitis, Vytenis-Pranas Deltuva, Daina Skiriutė

**Affiliations:** 1 Laboratory of Molecular Neurooncology, Neuroscience Institute, Lithuanian University of Health Sciences, Eiveniu str. 4, 50161 Kaunas, Lithuania; 2 Laboratory of Biophysics and Bioinformatics, Neuroscience Institute, Lithuanian University of Health Sciences, Eiveniu str. 4, 50161 Kaunas, Lithuania

**Keywords:** CNS cancer, Biomarkers

## Abstract

Astrocytoma is the most common glial tumour of the CNS. The most malignant form is grade IV Astrocytoma, also called Glioblastoma. Due to its heterogeneity, aggressiveness and lethal nature scientists are trying to find less invasive methods for early prediction of tumour onset, recurrence, response to therapy and patients’ survival. Here, applying decision tree classification algorithm we performed astrocytoma specific protein profile analysis on serum proteins TIMP-1, active and latent form of TGF-β1, IP-10, ANGPT-1, OPN, and YKL-40 using enzyme-linked immunosorbent detection assay (ELISA). Results have demonstrated that astrocytoma specific profile consisted of three proteins—active form of TGF-β1, TIMP-1 and YKL-40 and was able to correctly classify 78.0% (103/132) of sample and 83.3% (60/72) of astrocytoma sample. Calculating decision tree algorithm associated with astrocytoma patient survival, prediction model reached an accuracy of 83.3% (60/72). All together these results indicate that glioma detection and prediction from patient serum using glioma associated proteins and applying mathematical classification tools could be achieved, and applying more comprehensive research further could be implemented in clinic.

## Introduction

Astrocytoma is the most common type of brain tumour originating in astrocytes, which are star-shaped brain cells located in the cerebrum^1^. According to the World Health Organization (WHO) classification, based on histological parameters, astrocytoma is divided into grade II (diffuse astrocytoma), grade III (anaplastic astrocytoma) and grade IV (glioblastoma (GBM))^2^. A higher grade astrocytoma represents a worse prognosis and is more aggressive. Patients with grade II astrocytoma have a median survival of ~ 7–8 years. While patients with grade III astrocytoma have a median survival of ~ 2–3 years, and patients with GBM of only 9–14 months^3^. Delayed diagnosis is a major contributing factor to lower patients‘ survival. Mostly, astrocytoma is diagnosed by computer tomography or magnetic resonance imaging scan. Although these methods are reliable and used for many years, they are expensive and usually used after the tumour has spread a lot^4^. Therefore, it is important to find new non-invasive methods that could detect tumour in early stages and prognosticate its progression, and response to therapy. One of the most promising non-invasive methods could be detection of certain molecules, i.e. biomarkers, such as proteins in the patient’s blood, however as to our knowledge none is used to diagnose astrocytoma yet.

A recent study done by our group^5^ revealed promising blood serum candidate protein markers of astrocytoma progression. We constructed decision tree classification algorithm for the indication of presence of astrocytoma—specific serum protein profiles, and found that tissue inhibitor of matrix metalloproteinase 1 (TIMP-1), active transforming growth factor beta 1 (TGF-β1), C-X-C motif chemokine ligand 10 also known as interferon gamma-induced protein 10 (IP-10), angiopoietin-1 (ANGPT-1) and osteopontin (OPN) can be potential mediators of astrocytoma progression. In addition, several studies have confirmed that these proteins could be potential astrocytoma diagnostic biomarkers due to their biological functions during oncogenesis^6–11^. Besides, recently numerous studies emphasized chitinase-3-like protein 1 (CHI3L1), also known as YKL-40 as one of the most promising astrocytoma diagnostic and prognostic biomarker^12–14^. To strengthen the proposed model, we decided to include YKL-40. Additionally, model was supplemented with analytes from brain tumour meningioma in order to check model specificity to glioma. Since the majority of proteins are implicated in many different biological processes it is important to investigate sorted out protein’s cancer-type specificity (i. e. glioma) comparing to cancers having different type of pathogenesis mechanism. Meningioma is dural-based tumour that arise from arachnoid cap or meningothelial cells^15^ and shares the same location as glioma, while yet having distinct nature, meningioma could serve as convenient model to study molecular markers specificity to glioma.

Here we performed seven serum proteins—TIMP-1, active and latent forms of TGF-β1, IP-10, ANGPT-1, OPN, and YKL-40—analysis in astrocytic glioma patient sample applying conventional ELISA assay. The aim of this study was the construction of the diagnostic and prognostic glioma pathology associated serum protein profiles applying decision tree method.

## Results

### Protein levels in different brain tumour entities

**Figure 1:**
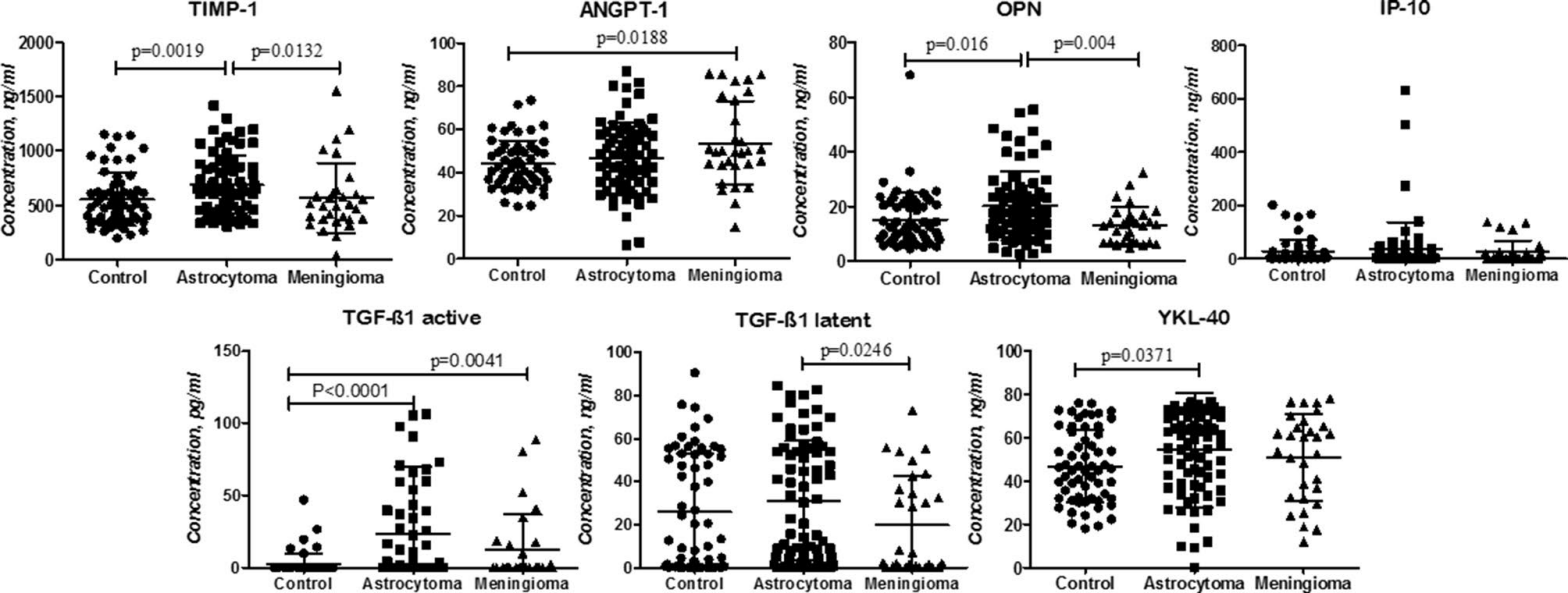
TIMP-1, ANGPT-1, OPN, IP-10, TGF-β1 active, TGF-β1 latent and YKL-40 proteins expression levels in astrocytoma (grade II-IV) patients’, healthy control and benign meningioma patients’ peripheral blood serum. Middle line in each group represents mean, lower and upper lines—standard deviation (SD).

Protein expression levels in astrocytoma and meningioma patient blood serum using ELISA method were investigated. The expression levels among different brain tumours (astrocytoma (grade II-IV) vs meningioma (grade I)) and control group are demonstrated in Fig. 1. TIMP-1, OPN, YKL-40, and active form of TGF-β1 protein levels in astrocytoma serum substantially differed from levels in healthy control group. TIMP-1, OPN, and latent form of TGF-β1 levels in astrocytoma differed from meningioma group. ANGPT-1 as well as active form of TGF-β1 demonstrated difference between healthy control and meningioma group.

### mRNA expression of protein coding genes in tumour tissue

To elucidate if the protein level in blood serum is related with the presence of brain tumours, we investigated the mRNA expression of *TIMP-1, ANGPT1, SPP1* (OPN coding gene), *IP-10, TGF-β1* and *TIMP-1* in astrocytoma samples as well as in healthy human brain tissue.

**Figure 2:**
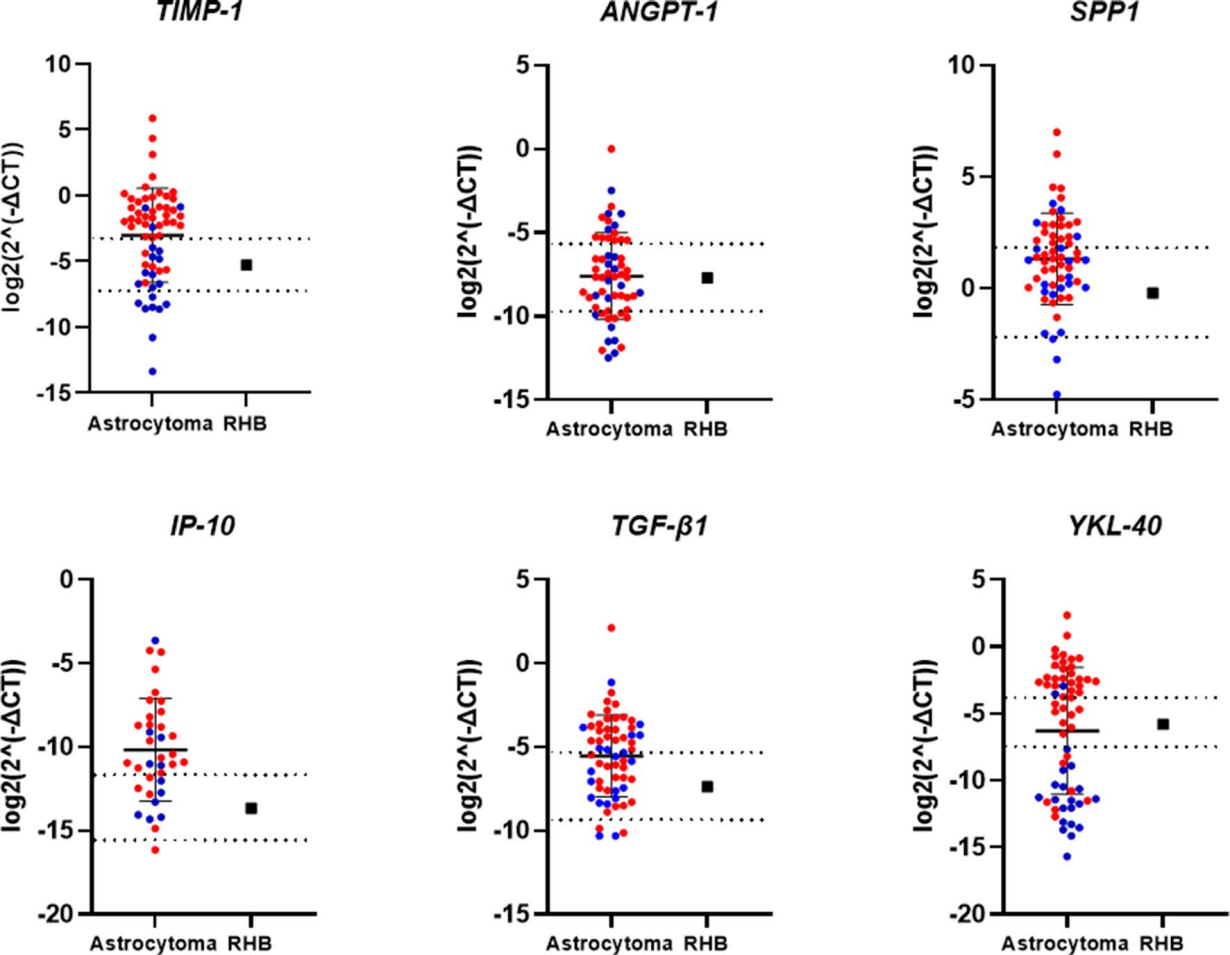
mRNA level of *TIMP-1, ANGPT1, SPP1, IP-10, TGF-β1* and *TIMP1* genes in astrocytoma and healthy human brain specimens. RHB—reference human brain RNA sample (pool of RNA from 12 healthy individuals). Blue dots in astrocytoma group indicates grade II astrocytoma samples, and red dots—samples of GBM. The upper and lower dotted lines indicate twofold higher and twofold lower mRNA expression points from RHB, respectively. Middle line in astrocytoma group represents mean, lower and upper lines—standard deviation (SD).

In total mRNA expression of *TIMP-1, ANGPT1, SPP1, TGF-β1* and *YKL-40* genes was analysed in 61 patients with different grade astrocytoma: 20 grade II diffuse astrocytoma, and 41 grade IV astrocytoma (glioblastoma), and in case of *IP-10—*in 11 grade II diffuse astrocytoma, and 24 glioblastoma samples. All genes mRNA level was quantified in tumour tissue using qRT-PCR, and compared with mRNA level in Human normal brain RNA sample “FirstChoice Human Brain Reference RNA” (Ambion), which was a pool of RNAs assembled from 12 donors from several brain regions, as described by the manufacturer. qRT-PCR showed stronger expression of *SPP1*, and *TGF-β1*, in most astrocytoma samples of both grade II and IV compared to normal brain sample. Elevated expression only in GBM samples compared to normal brain was visible in *TIMP-1, IP-10*, and *YKL-40* gene groups. *YKL-40* also shown downregulated expression in grade II astrocytoma tumour samples compared with normal brain tissue (Fig. 2). These data are in the line with one of protein expression in serum described above.

### Combined Analysis of Candidate Protein Levels in Astrocytoma Serum

**Figure 3:**
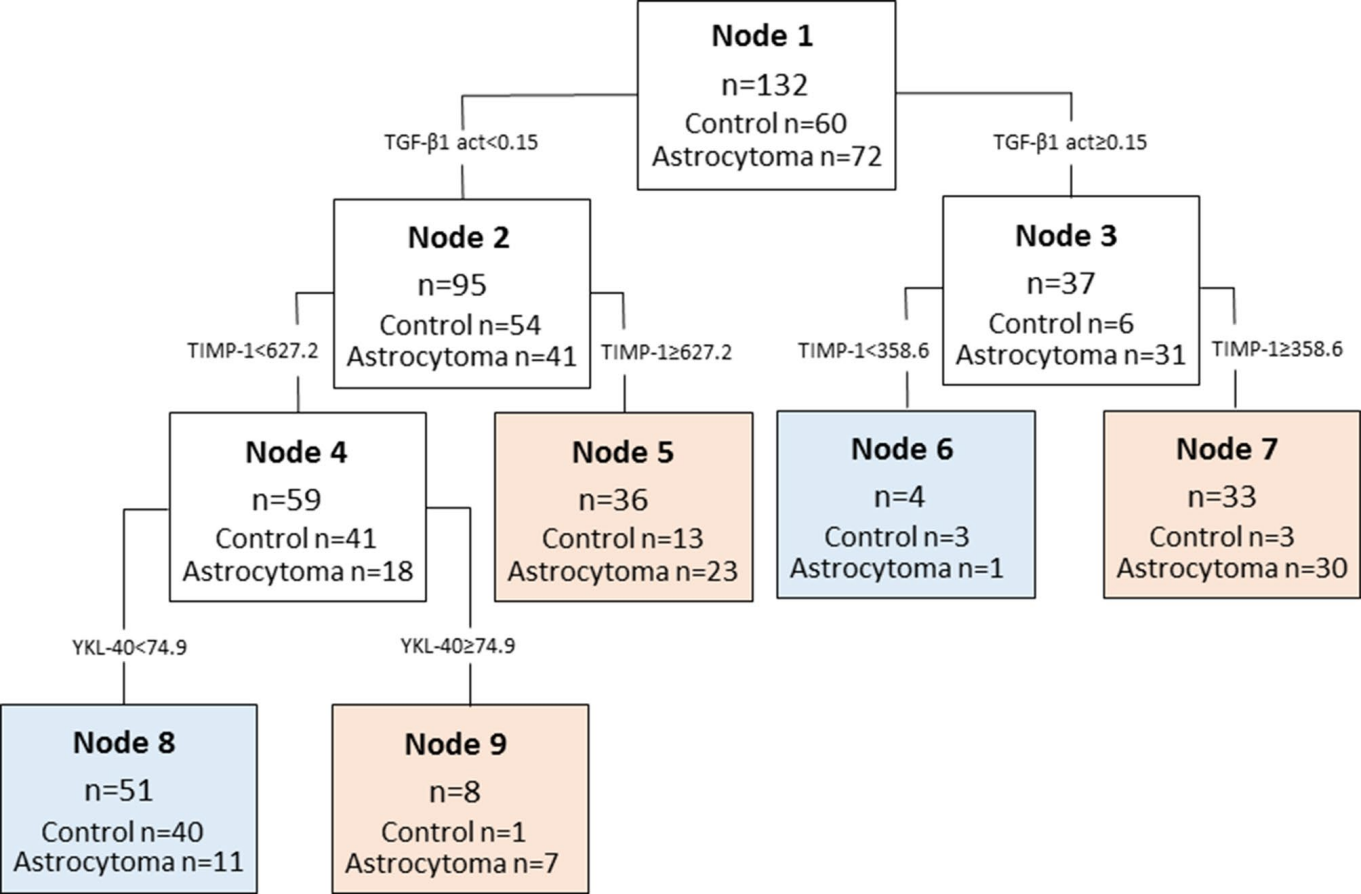
Diagnostic astrocytoma decision tree model, composed of active (act) TGF-β1, TIMP-1 and YKL-40 proteins in astrocytoma and healthy control serum. Tree was calculated from the results of 132 participants (60—controls and 72—astrocytomas). The number of all participants assigned to the specific class is represented by n, below—the number of participants in healthy control and astrocytoma group. Rose colour nodes represent classes, which predict participant having astrocytoma, blue nodes—healthy participant.

The decision tree classification analysis was performed using “Classification Learner” application provided by MathWorks MatLab on TIMP-1, ANGPT-1, OPN, IP-10, TGF-β1 active, TGF-β1 latent and YKL-40 protein expression levels in astrocytoma patients and healthy control groups. The results demonstrated classification tree composed of three proteins—active TGF-β1, TIMP-1, YKL-40, and correctly classified 78.0% (103/132) of the whole sample, 71.7% (43/60) of healthy control sample and 83.3% (60/72) of astrocytoma sample (Fig. 3).

Decision tree models were also applied using different tumour entities as variables, however, none of the models achieved higher accuracy than the previous one. Model of healthy group vs. patients with any type of brain cancer demonstrated 72.8% accuracy, healthy control vs. grade II astrocytoma vs. GBM—68.9%; healthy control vs. astrocytoma (grade II-IV) vs. meningioma—63.0%; healthy control vs. benign brain tumours (grade I meningioma and grade II astrocytoma) vs. GBM—56.8%, healthy control vs. GBM—75.8%; healthy control vs. grade II astrocytoma vs. meningioma—76.5%.

### Decision tree model specificity to brain tumours

As earlier mentioned, meningioma in this study serves as a model to investigate the specificity of glioma molecular markers. Herein, we tested the algorithm’s calculated for astrocytoma detection from blood serum and composed of active TGF-β1, TIMP-1 and YKL-40, ability to differentiate meningioma. Half of meningioma patients (15/30) were assigned to healthy control group, while another half—to astrocytoma group. Altogether, decision tree model for healthy control vs. astrocytoma (grade II-IV) vs. meningioma reached 63.0% accuracy with only 13.3% (4/30) of correct samples assigned to meningioma group, indicates model selectivity to glioma type cancer.

### Survival analysis

**Figure 4:**
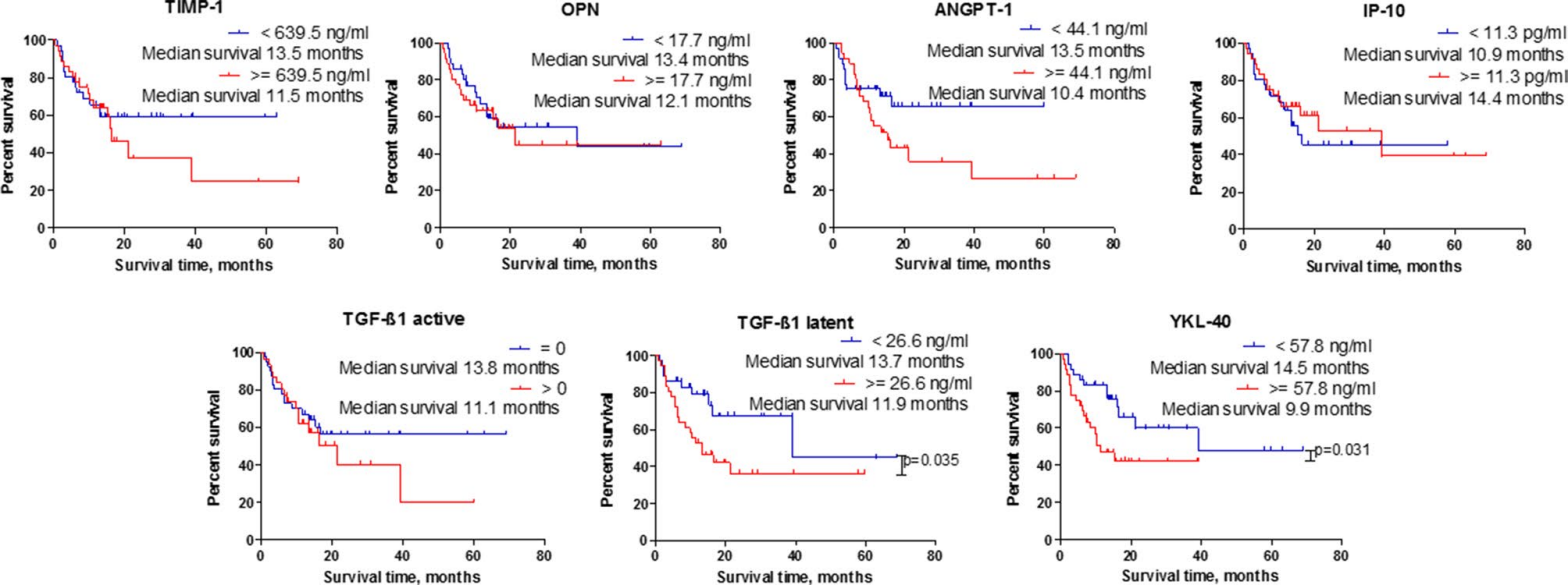
Survival dependency on TIMP-1, OPN, ANGPT-1, IP-10, active and latent TGF-β1, and YKL-40 proteins concentration in astrocytoma patients peripheral blood serum. TIMP-1 (n = 72) low (< 639.5 ng/mL) versus high (≥ 639.5 ng/mL) protein level patient group with median survival of 13.5 months versus 11.5 months, respectively; log-rank test; x^2^ = 0.535; df = 1; p = 0.464; OPN (n = 72), low (< 17.7 ng/mL) versus high (≥ 17.7 ng/mL) protein level patient group with median survival of 13.4 months versus 12.1 months, respectively; log-rank test; x^2^ = 0.231; df = 1; p = 0.631; ANGPT-1 (n = 72), low (< 44.1 ng/mL) versus high (≥ 44.1 ng/mL) protein level patient group with median survival of 13.5 months versus 10.4 months, respectively; log-rank test; x^2^ = 2.679; df = 1; p = 0.102; IP-10 (n = 72), low (< 11.3 pg/mL) vs. high (≥ 11.3 pg/mL) protein level patient group with median survival of 10.9 months versus 14.4 months, respectively; log-rank test; x^2^ = 0.293; df = 1; p = 0.589; active TGF-β1 (n = 72), low (0 pg/mL) versus high (> 0 pg/mL) protein level patient group with median survival of 13.8 months versus 11.1 months, respectively; log-rank test; x^2^ = 0.617; df = 1; p = 0.432; latent TGF-β1 (n = 72), low (< 26.6 ng/mL) versus high (≥ 26.6 ng/mL) protein level patient group with median survival of 13.7 months versus 11.9 months, log-rank test; x^2^ = 4.468; df = 1; p = 0.035; YKL-40 (n = 72), low (< 57.8 ng/mL) versus high (≥ 57.8 ng/mL) protein level patient group with median survival of 14.5 months versus 9.9 months, respectively; log-rank test; × 2 = 4.672; df = 1; p = 0.031; Blue colour—low protein level, red color—high protein level.

Kaplan–Meier survival analysis demonstrated that astrocytoma patients with higher than median YKL-40 and latent form of TGF-β1 levels in blood serum survived significantly longer (p = 0.031 and p = 0.035, respectively), while the lower than median protein concentration in patient serum indicated shorter patients’ survival. TIMP-1, ANGPT-1 and active form of TGF-β1 demonstrated the tendency of patient survival association with the lower protein level in astrocytoma, but the difference was not significant. IP-10 and OPN haven’t shown any associations (Fig. 4). Survival analysis in separate GBM group for studied serum proteins did not showed any differences (see Supplementary Fig. S1).

**Figure 5:**
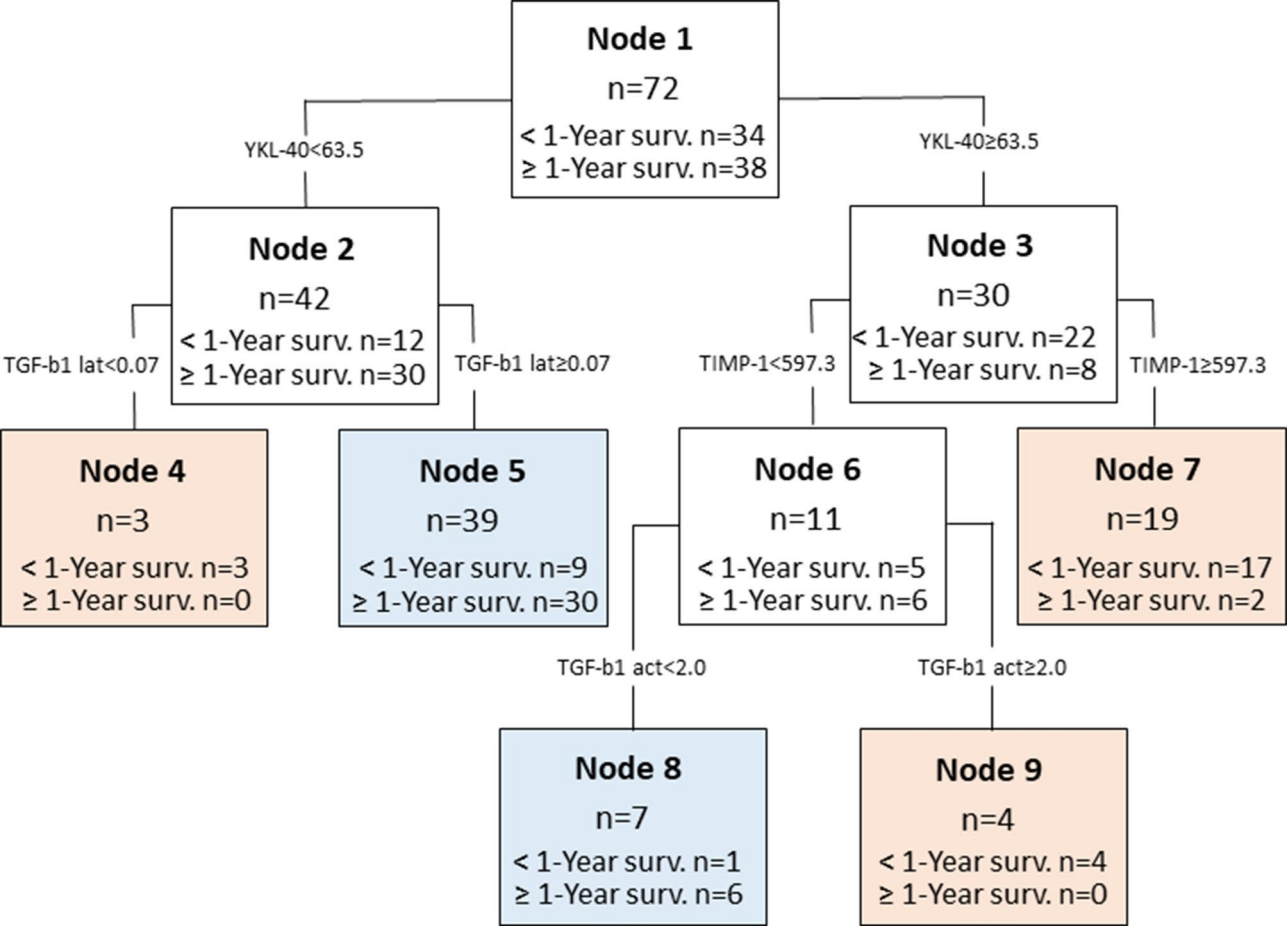
Potential prognostic profile of serum proteins YKL-40, latent (lat) and active (act) forms of TGF-β1 and TIMP-1 for astrocytoma patients. The overall accuracy of the algorithm reached 83.3% (60/72), less than one year survival accuracy reached 70.6% (24/34), while more than 1 year survival accuracy reached 94.7% (36/38). The number of participants assigned to the specific class is represented by n. Rose colour represents classes, predicting shorter than a year patient survival, blue—longer than a year patient survival group.

In the combined survival analysis, total (n = 72) astrocytoma sample was divided into two groups—patients who survived less than a year after the surgery (n = 34), and those who survived longer than a year (n = 38). Calculated decision tree algorithm with the highest accuracy of 83.3% (60/72) consisted of YKL-40, latent and active TGF-β1 and TIMP-1 and predicted the survival of more than one year with a specificity of 94.7% (36/38), while shorter than a year survival—with a specificity of 70.6% (24/34) (Fig. 5.).

## Discussion

In decades of glioma research none blood serum or plasma marker for disease detection or prediction have been introduced to clinic, on the other hand, many studies have tried to find single marker or complexes, which could be specific enough to recognize glioma tumour from blood. In this study the potential of blood serum proteins for detection and prediction of astrocytoma was tested. Promising candidate proteins—TIMP-1, ANGPT-1, OPN, IP-10, active form of TGF-β1, latent form of TGF-β1 and YKL-40—were chosen according to the earlier our laboratory findings^5^ and broad literature review^6–14,16–19^. Here we showed that protein levels of TIMP-1, OPN, YKL-40, and active form of TGF-β1 in astrocytoma patient serum substantially differed as compared to control (healthy) group, while the differences between healthy control and meningioma group were observed only for ANGPT-1 and TGF-β1 active. Altogether these results reiterate numerous other studies. Tissue inhibitor of metalloproteinases (TIMPs) together with matrix metalloproteinases (MMPs) regulates degradation of extracellular matrix (ECM) and are very important factors in tumour invasion processes. Lin and colleagues showed that TIMP-1 level was increased in glioma patient's plasma as compared to normal controls^19^. Next protein in our investigation is OPN well known as a modulator of apoptosis, extracellular matrix degradation, cell migration and neovascularization^20^. Elevated levels of OPN in patient blood have been reported in many cancer types^21^ as well as in astrocytoma^16^. YKL-40 has been considered as one of the most promising proteins in astrocytoma detection. It is 40-kDa secreted glycoprotein, which is produced by cancer cells and is involved in many cancer related processes like cell proliferation, apoptosis, differentiation, angiogenesis, and regulation of extracellular tissue remodelling^12,13^. The differences in YKL-40 protein serum level between healthy subjects and glioma patients have been demonstrated already in 2002 by Tanwar group^13^, and a comprehensive analysis of Zhi-Qiang Li^14^ group confirmed YKL-40 protein being a valuable biomarker for glioma patient prognosis. Next protein in our analysis, namely TGF-β1 in blood serum can be measured in two forms—latent and active. Together with Latency Associated Peptide (LAP) the mature TGF-β1 dimer form a latent complex, in which TGF-β1 is unable to bind its receptors. In a few cell types, latent TGF-β1 can be activated, when release from the LAP occurs. Activation of the cytokine implies release of mature TGF-β1 from LAP^22^. Dysregulated TGF-β signalling was reported in many patients, and is possibly related with initiation and progression of many cancers including glioma^9^. As to our knowledge, none of these proteins are reported to associate with meningioma as possible serum molecular marker, however abnormal levels of TIMP-1 and OPN have been reported in meningioma tumour tissue^23,24^.

Herein, as most promising protein profile for both low and high grade astrocytoma detection was composed of TGF-β1 active, TIMP-1 and YKL-40. Decision tree algorithm correctly classified 78.0% (103/132) of the sample, 71.7% (43/60) of healthy controls sample and 83.3% (60/72) of astrocytoma sample. Interestingly, fibrous meningioma sample following this model were equally assigned to both healthy control and astrocytoma groups, indicating probable protein profile specificity to glioma cancer, rather than brain cancer in common.

Earlier we demonstrated^5^ decision tree which consisted of ANGPT-1, TIMP-1, IP-10 and active TGF-β1 proteins, algorithm reached accuracy of 73.5% (75/102) and correctly classified 79.7% (47/59) of all astrocytomas and 65.1% (28/43) of all healthy controls. Accuracy of the decision tree calculated using expression levels of those four proteins in the independent sample set demonstrated even greater results: the accuracy of 75.8%, with 100 of 132 correctly assigned participants (healthy vs. astrocytoma patients) to the right group (see Supplementary Fig. S2). Furthermore, the model demonstrated glioma type cancer specificity as 53.3% (16/30) of fibrous meningioma samples were assigned to control group, while 47.7% (14/30)—to astrocytoma group using the same decision tree parameters. Elstner with colleagues proposed promising serum protein profile, consisting of BMP2, HSP70, and CXCL10 (IP-10), which was able to distinguish GBM group with the specificity and sensitivity of 89% and 96% from control one, respectively^25^. Pérez‐Larraya in 2014 published 2-step diagnostic algorithm using glial fibrillary acidic protein (GFAP) and YKL‐40 which differentiated patients with GBM from those with nonglial brain tumours with a sensitivity of 65% and a specificity of 78%^26^.

Despite many innovations in the modern medicine and combined treatment applying surgery, chemotherapy and radiotherapy, brain gliomas remain lethal, aggressively growing tumours leading to short patient survival. The possibility almost noninvasively prognosticate glioma patient survival could help to optimize tumour treatment decisions. Here we presented decision tree model which was able to correctly predict 60 out of 72 astrocytoma patients (83.3%) one year survival with the accuracy of 94.7% (36/38). Furthermore, prediction tree consisted of the same proteins as the astrocytoma detection algorithm that is TGF-β1 active, TIMP-1, YKL-40, with the addition of the protein of latent form TGF-β1. Several promising protein profiles prognosticating patient survival were also demonstrated earlier. The group of Lin have shown that the panel of four cytokines—IL-7, IL1R4/ST2, sgp130 and MCP-1 substantially correlated with GBM patient's overall survival^27^, as well as Elstner and his group proposed the protein profile formed of TSP1, HSP70, and IGFBP3, which was associated with more than 15 months survival after GBM tumour resection^25^.

All together these results indicate that glioma detection and prediction from patient serum using glioma associated proteins and applying mathematical classification tools could be achieved and further comprehensive research could help to implement molecular prediction tools into clinical usage.

## Materials and methods

### Study group and blood collection

Study group was collected from 2014 to 2019 at the Neurosurgery department of the Hospital of Lithuanian University of Health Sciences Kaunas Clinics with the informed consent of all participants. Investigation methodologies were approved by Kaunas Regional Biomedical Research Ethics Committee (No. P2-9/2003) and were carried out under the Declaration of Helsinki. In the study group of 162 serum samples 72 were histopathologically confirmed astrocytoma (12 of which—grade II astrocytoma; 60 of which—grade IV astrocytoma), 30 samples fibrous meningioma (benign brain tumour) and 60 control samples from healthy volunteers. Median/mean age of healthy control group was 51.7/51.8 yr., median/mean age of astrocytoma group—62.9/58.1 yr. and median/mean age of meningioma group was 61.2/62.9 yr. Blood from both astrocytoma and meningioma patients were collected before tumour resection and any kind of treatment, while after operation the standard treatment was applied (radiotherapy plus chemotherapy). For control group healthy volunteer individuals with no evidence of brain or any other type of cancer along with no indications of inflammation or infection were chosen. Collected blood serum samples were allowed to clot from 30 min. to 1 h at RT and then were centrifuged at 1300xg for 10 min. Serum was collected, transferred to fresh Eppendorf tubes and stored at—80 °C until further investigation stage.

### Evaluation of serum protein levels

For protein concentration measurements in participant’s blood serum R&DSystems ELISA kits were used: for TIMP-1—DY970-05 kit, for ANGPT-1—DY923 kit, for OPN—DY1433 kit, for YKL-40—DY2599 kit, both forms of TGF-β1—DY240-05 kits and for IP-10—DY266-05 kit. For each protein detection, different dilution factor was used according to the manufacturers’ recommendations, other groups studies, or pilot experiments^28–33^. Serum for TIMP-1 was diluted 400 folds; ANGPT-1—20 folds; OPN—20 folds; YKL-40—50 folds; TGF-β1 active—undiluted; TGF-β1 latent—undiluted; IP-10—undiluted. To avoid multiple freeze thaw cycles before the first experiment each serum sample was aliquoted according to chosen dilution factor for every investigated protein, including two additional tubes with serum quantity equal to one undiluted sample in case of unsuccessful experiment and stored at—80 °C until further investigation stage. Every sample was measured in duplicate. Protein concentration of TGF-β1 in serum was measured for both active ant latent forms. All experimental steps and data analysis were performed according to the manufacturers’ recommendations^28–33^. Optical densities were measured at a wavelength of 450 nm using a “Sunrise” absorbance reader (“Tecan Trading”, Switzerland).

### RNA extraction, cDNA synthesis and qRT-PCR

RNA was extracted from 100 mg of nitrogen frozen tumor tissue using “mirVana miRNA Isolation Kit” (Invitrogen, USA). cDNA was synthesized from 2 µg RNA using “High-Capacity cDNA Reverse Transcription Kit” (Applied Biosystems, USA) with Ribolock RNase inhibitor. Reverse transcription was performed with hexamer primers and MultiScribe Reverse Transcriptase according to manufactures instructions. PCR applying Sybr-Green I (“Maxima SYBR Green/ROX qPCR Master Mix”, Thermo Scientific, Lithuania) assay was carried out in 12 µl of total reaction volume including 15 ng of cDNA. Primers were designed using “PerlPrimer3” and synthesized by Metabion International (Germany). Primer sequences could be obtained under request. qRT-PCR was performed on AB7500 fast platform (Applied Biosystems). Gene expression level was calculated applying dCt method and geometric mean of ACTB and GAPDH Ct values was used for data normalization.

### Statistical analysis

To analyse gathered data, software packages GraphPad Prism version 6.0 (San Diego, CA, USA) and SPSS Statistics 19 (Chicago, IL, USA) were used. First, distribution of data sets were verified using Kolmogorov–Smirnov test. To evaluate differences of each investigated protein levels in blood serum between two independent groups, both with normally distributed data parametric Student’s t-test was applied. To compare groups, where one or both data sets are abnormal the non-parametric Mann–Whitney test was used. Kaplan–Meier method was used to estimate astrocytoma patient survival function. All analysed protein groups were divided into lower (< median value) and higher (≥ median value) protein expression groups, according to the median values of each protein level and the log-rank test was used to compare the survival curves. Survival time was calculated starting from the date of operation until death, or the date of project closure.

For Decision Tree analysis was applied “Classification Learner” application provided by MathWorks as part of Machine Learning Toolbox in MATLAB^34^.

## Supplementary Information

Supplementary Information.

## Data Availability

The datasets generated during and analysed during the current study are available from the corresponding author on a request.
