# Supplementary material for: Serum protein triplet TGF-β1, TIMP-1, and YKL-40 serve as diagnostic and prognostic profile for astrocytoma
Source: Sci Rep. 2021 Jun 23;11:13100. doi: 10.1038/s41598-021-92328-3 (PMC8222249; doi:10.1038/s41598-021-92328-3)
Supplement: Supplementary file 1 — Supplementary Information. [file 41598_2021_92328_MOESM1_ESM.pdf]

Serum Protein Triplet TGF-β1, TIMP-1, and YKL-40 Serve as Diagnostic and Prognostic Profile for Astrocytoma

Rūta Urbanavičiūtė[1], Rūta Zabitaitė[1], Algimantas Kriščiukaitis[2], Vytenis-Pranas Deltuva[1], Daina Skiriutė[1]

[1] Laboratory of Molecular Neurooncology, Neuroscience Institute, Lithuanian University of Health Sciences, Eiveniu str. 4, LT50161 Kaunas, Lithuania;

[2] , Laboratory of Biophysics and Bioinformatics, Neuroscience Institute, Lithuanian University of Health Sciences, Eiveniu str. 4, LT50161 Kaunas, Lithuania;

* Correspondence: ruta.urbanaviciute@lsmuni.lt; Phone.: +370 37 326769

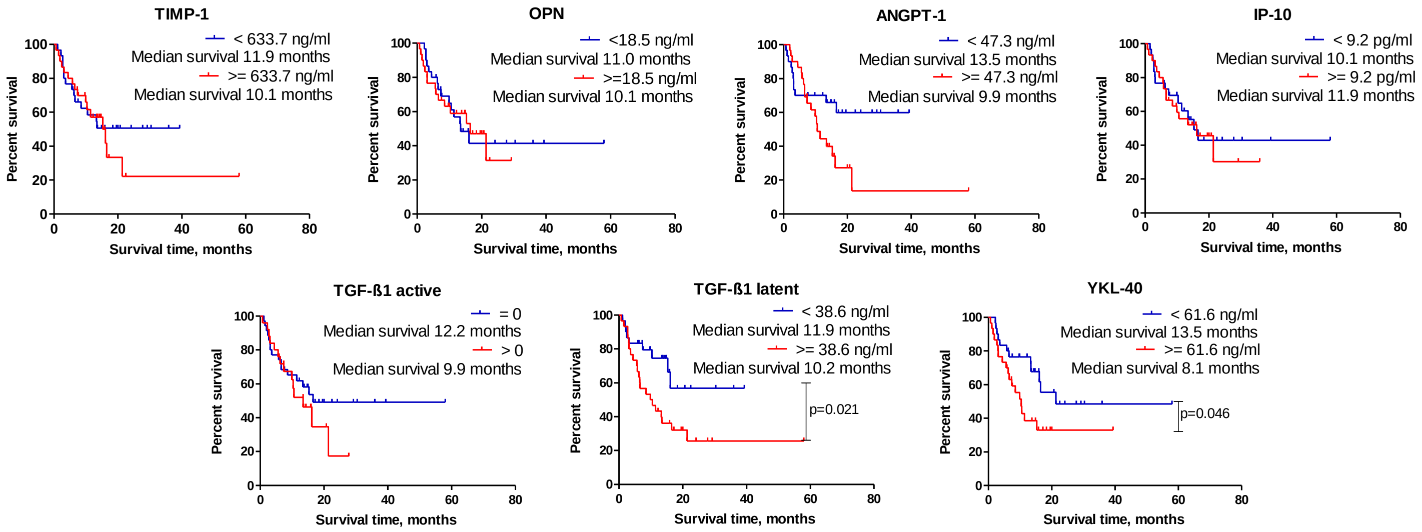

Figure S1. Survival dependency on TIMP-1, OPN, ANGPT-1, IP-10, active and latent TGF-β1, and YKL-40 proteins concentration in glioblastoma patients peripheral blood serum. TIMP-1 (n = 60), low (< 633.7 ng/mL) vs. high (≥ 633.7 ng/mL) protein level patient group with median survival of 11.9 months versus 10.1 months, respectively; log-rank test; $x^2 = 0.306$; df = 1; p = 0.580; OPN (n = 60), low (< 18.5 ng/mL) vs. high (≥ 18.5 ng/mL) protein level patient group with median survival of 11.0 months versus 10.1 months, respectively; log-rank test; $x^2 = 0.015$; df = 1; p = 0.902; ANGPT-1 (n = 60), low (< 47.3 ng/mL) vs. high (≥ 47.3 ng/mL) protein level patient group with median survival of 13.5 months versus 9.9 months, respectively; log-rank test; $x^2 = 3.502$; df = 1; p = 0.061; IP-10 (n = 60), low (< 9.2 pg/mL) vs. high (≥ 9.2 pg/mL) protein level patient group with median survival of 10.1 months versus 11.9 months, respectively; log-rank test; $x^2 = 0.102$; df = 1; p = 0.749; active TGF-β1 (n = 60), low (0 pg/mL) vs. high (> 0 pg/mL) protein level patient group with median survival of 12.2 months versus 9.9 months, respectively; log-rank test; $x^2 = 0.843$; df = 1; p = 0.359; latent TGF-β1 (n = 60), low (< 38.6 ng/mL) vs. high (≥ 38.6 ng/mL) protein level patient group with median survival of 11.9 months versus 10.2 months, respectively; log-rank test; $x^2 = 5.308$; df = 1; p = 0.021; YKL-40 (n = 60), low (< 61.6 ng/mL) vs. high (≥ 61.6 ng/mL) protein level patient group with median survival of 13.5 months versus 8.1 months, respectively; log-rank test; x2 = 3.967; df = 1; p = 0.046; Blue colour – low protein level, red colour – high protein level.

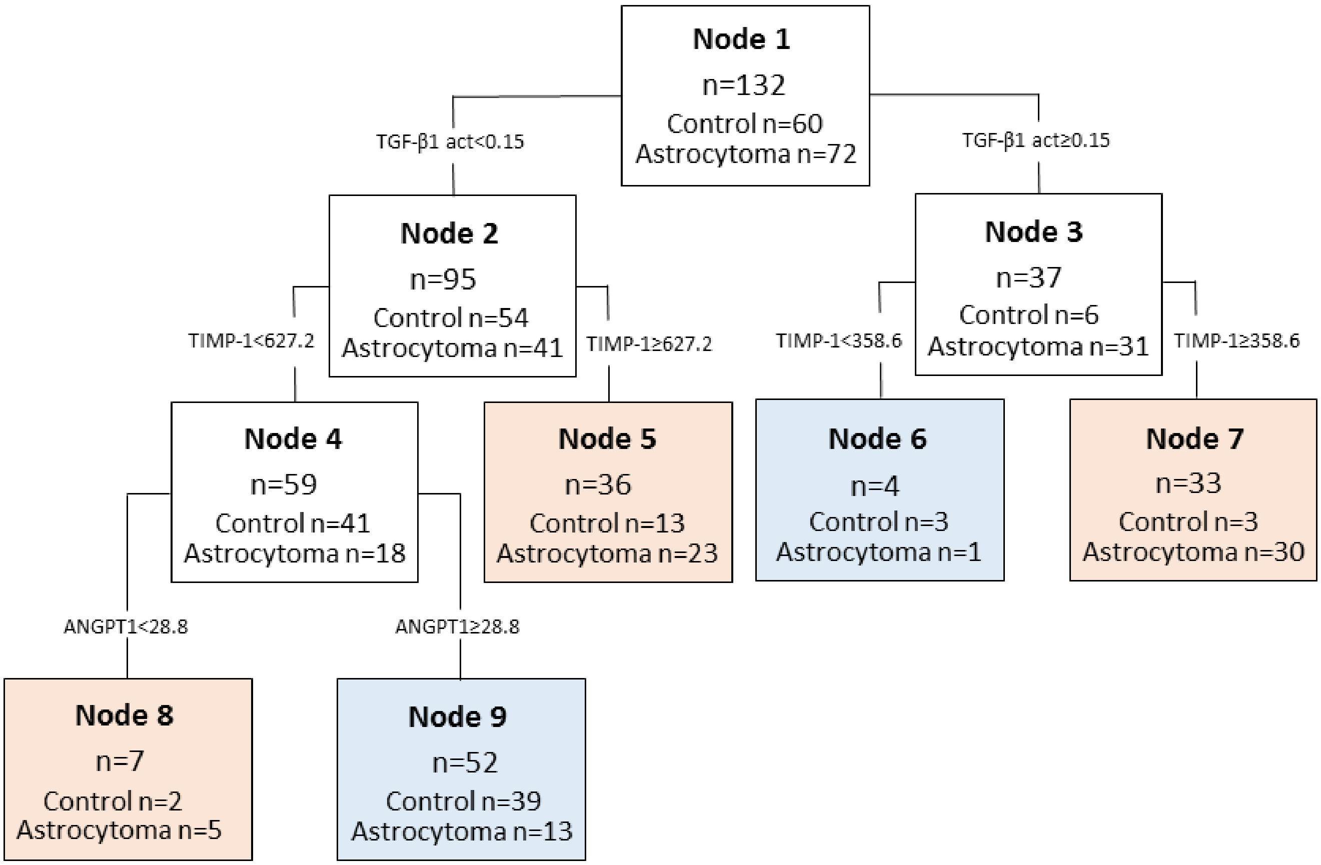

Figure S2. Astrocytoma detection tree model, composed of active (act) TGF-β1, TIMP-1 and ANGPT-1 proteins in astrocytoma and healthy control serum. Tree was calculated from the result of 132 participants (60 – controls and 72 - astrocytomas). The number of all participants assigned to the specific class is represented by n, below – the number of participants in healthy control and astrocytoma group. Rose colour nodes represent classes, which predict participant having astrocytoma, blue nodes – healthy participant.
